# Healthcare needs of people living with human immunodeficiency virus: A qualitative descriptive study

**DOI:** 10.1002/nop2.1071

**Published:** 2021-09-23

**Authors:** Kusman Ibrahim, Yusshy Kurnia Herliani, Laili Rahayuwati, Siti Khadijah, Titin Sutini

**Affiliations:** ^1^ Departement of Medical and Surgical Nursing Faculty of Nursing Universitas Padjadjaran Bandung Indonesia; ^2^ Departement of Community Health Nursing Faculty of Nursing Universitas Padjadjaran Bandung Indonesia; ^3^ Departement of Mental Health Nursing Faculty of Nursing Universitas Padjadjaran Bandung Indonesia

**Keywords:** health care, HIV infections, needs, qualitative

## Abstract

**Aim:**

A better understanding about the health care and support needs is beneficial to maintain the linkage between People Living with Human Immunodeficiency Virus (PLWH) and healthcare services. This study aims to explore the healthcare needs of PLWH from their perspectives.

**Design:**

A qualitative descriptive study design was undertaken in July 2017 to June 2018.

**Methods:**

Fourteen participants were recruited by purposive sampling technique. Face‐to‐face in‐depth interview and focus‐group discussion (FGD) were conducted and analysed using content analysis.

**Results:**

Data revealed five themes, namely the needs to be free from stigma and discrimination, strengthen life spirit, have safe sexual practices, social support, and have accessible and affordable healthcare services.

**Conclusion:**

People Living with Human Immunodeficiency Virus have their own perspectives on their healthcare needs. Therefore, nurses and other healthcare providers need to explore, understand, and respond to the needs, and incorporate them into comprehensive and holistic care.

## 1 INTRODUCTION

The ambitious target to end Human Immunodeficiency Virus (HIV) epidemic by 2030 requires serious attention, strong commitment, and effective interventions to mitigate and stop new transmission, as well as to treat and recover those who are already infected. Indonesia is the most populated country in South‐East Asia region with about 640,000 people living with HIV (PLWH) infection in 2018, followed by Thailand 480,000, Myanmar 240,000, and Nepal 30,000 (UNAIDS, 2019). Although the national prevalence of new cases has decreased from 63,000 (2010) to 43,000 (2018), the proportion of young people among new HIV infection is the third highest number in Asia and Pacific region. In addition, the number of Acquired Immunodeficiency Syndrome (AIDS)‐related deaths was increasing since 2010 to 2018 (UNAIDS, 2019). However, linkage to treatment and care services was poor, and more than half of people who knew their HIV status were not accessing antiretroviral therapy in 2018. In many sites, the percentages of patients with HIV loss to follow up were reported significant and ranged between 14% and 30% (Frijters et al., 2020; Haroen et al., 2017; Widyanthini et al., 2014).

Therefore, retention in care becomes a crucial issue for the success of treatment. In a general term, this retention refers to a patient's regular engagement with healthcare services after initial entry into the service (Roscoe & Hachey, 2020). Meanwhile, the Center for Disease Control and Prevention (CDC) reported retention in care among PLWH in United States from 2010 to 2016 and ranged from 53.6% to 57.6% (CDC, 2019). In Nigeria, a study found that the retention in care was about 81.1% in private hospitals and 80.3% in public hospitals (Umeokonkwo et al., 2019). An observational prospective cohort study in four places in Indonesia (Bandung, Jakarta, Yogyakarta, Bali) found that retained in care among PLWH was 75.4%, however only 35% had virally suppressed, and 24% of whom were loss to follow up (Januraga et al., 2018). Furthermore, some studies identified that several factors might be associated with retention in care, which includes patients’ beliefs, providers‐patients’ interaction, medication side effects, stigma, lack of social support, distance, financial constraints, information, and resources availability (Lifson et al., 2017; Mukumbang et al., 2017; Nugroho et al., 2018).

### 1.1 Background

People Living with Human Immunodeficiency Virus face many challenges in their life starting from continuing life as an individual, interpersonal, social, and economic problems (Dulin et al., 2018). Not only that, living with HIV, they experienced discrimination and discrimination (Moussa et al., 2021; Sukartini et al., 2021; Ziersch et al., 2021) barriers while accessing healthcare services (Gogishvili et al., 2021), emotional state, and difficulties concerning spouse/partner notification issues (Senyurek et al., 2021). Healthcare needs that are needed by PLWH can include the needs of care, support, information, and acceptance from the community (Bernard et al., 2016; O’Loughlin et al., 2020). Several previous studies have shown that knowledge is an important requirement for PLWH to be able to choose the right and safe treatment. Besides that, mental health needs are also an important for PLWH (Agovi et al., 2020; Zunner et al., 2015).

Therefore, to maintain good engagement with healthcare services, the further study is essential to understand the characteristics and needs of the patients based on their own perspectives as PLWH. From behavioural perspective, patients can be seen as customers, and they would engage with a product or service when they could find a unique value of the services (Krist et al., 2017). To uncover the values and expectations of the patients, an explorative inquiry approach is needed. Therefore, this study aims to explore the healthcare needs of PLWH with qualitative approach, particularly in Indonesian setting and context.

## 2 METHODS

### 2.1 Study design

The study used a qualitative descriptive approach to describe healthcare needs among PLWH.

### 2.2 Participant selection and setting

Total of fourteen participants were recruited by purposive sampling from the HIV clinic of a major teaching hospital in West Java, Indonesia. The inclusion criteria were being adult, have been living with HIV for a minimum of 6 months after knowing their status (able to manage daily activity based on representative social theory) (Paschoal et al., 2014), and willing to participate in the study. Participants who cannot complete the study were excluded. From fourteen participants, eight participants were involved in the FGD session, and six others were individually in‐depth interviewed. In addition, the number of interviewed participants were determined by the data saturation level which is defined as the point when no new information is being generated in the data (Saunders et al., 2018).

### 2.3 Data collection

The study was conducted from July 2017 to June 2018. After obtaining permission from the hospital director, the researcher contacted a nurse in charge in the HIV Clinic. Through the nurse's assistance, respondents who met the criteria were selected and invited for the FGD session. The FGD was conducted to capture the overview of healthcare need, experience in accessing health care, and expectation towards services. Furthermore, the session was conducted in a convenient room nearby the clinic arranged by the researcher. The researchers explained the purpose, estimated time and expectations of the meeting and offer the participants to fill and sign the informed consent. After their permission, their voice was audiotape recorded during the discussion. A broad main question offered were “Please tell me why you need health care?” “can you talk about the barriers you feel?” and “can you talk about the other associated problems.” The participants were encouraged to feel free in responding to the question. The next participants could provide comments, opinions, or feeling based on their knowledge about the topic. Meanwhile, the facilitators’ role was to maintain that the discussion was running effectively, and all participants have equal chance to express their concerns and feelings about the given topic. The discussion lasted about two hours, and at the end of the session, the results were concluded, reward and appreciation were given to all participants for their value contribution.

Based on the FGD results, the researcher identified some issues that need to be followed up by interview, particularly about their feelings and experiences regarding special care needs. Meanwhile, six participants were selected to be separately in‐depth interviewed. Also, the researcher made appointment with the participants to have individual face to face interview base on the participants’ convenience time and setting. Some agreed to be interviewed after their visit to clinic was completed, while others were interviewed in their home. The interview lasted an average of about 60 min for each participant, and audiotape was recorded based on permission. In addition, each participant was interviewed at least twice including clarification and confirmation of the interview results to obtained appropriate data. Audio tape recorded data from FGD, and interviews were transcribed verbatim.

### 2.4 Trustworthiness

Trustworthiness of the data was assessed using the four criteria of credibility, dependability, transferability, and confirmability (Elo et al., 2014). Credibility was established by making initial data coding by two researchers, and then reviewed and reshaped by the other. Furthermore, selected interview transcripts were brought back to selected participants for clarification regarding meanings and interpretations made by the researcher. The participants agreed with the meanings and content of the transcript. Data were continuously assessed and evaluated for meaning and completeness to ensure its dependability. Confirmability was achieved by the classification of coding into categories and major categories was discussed together among all researchers. One researcher who has expertise in conducting qualitative study supervised and audited the whole process. Meanwhile, the combination of data collection method with FGD and in‐depth interview as well as purposive sampling of participants allowed deepness of the data. To improve transferability, a complete description about all steps of data collection and analysis and participants was explained.

### 2.5 Analysis

The analysis data were assisted by use of the NVIVO 12 software for organizing and coding the data. Then, the analysis of interview results used a content analysis (Bengtsson, 2016). The stages consisted of (1) decontextualization by identifying the meaning units; (2) recontextualization by including content and excluding dross; (3) categorization by identifying homogenous groups; and (4) compilation by drawing realistic conclusion.

### 2.6 Ethical consideration

This study was conducted after obtaining ethical clearance by the Research Ethic Committee Universitas Padjadjaran with the letter number 304/UN6.KEP/EC/2018. All the ethical issues of qualitative studies were considered. Before the data collection through in‐depth‐interview, the researchers explained the study purpose and confidentiality of the information was explained to the participants and written informed consent was obtained. In this study, the participants had the right to withdraw from the study at any time.

## 3 RESULTS

The FGD participants consisted of eight females and six males with age that ranged from 21 to 58 years. Four participants completed college educational background, while the rest completed senior and junior high school. Also, eight were self‐employed with irregular fix income, four were housewives, and the rest were unemployed. Nine participants were infected with HIV through heterosexual activity, while the rest from needle sharing as injecting drugs. In addition, they have been living with HIV since 2007 to 2017 years since knowing their status. The interview participants were selected from the FGD participant; therefore, their characteristics were covered as well in the FGD participant (Table 1).

**TABLE 1:** Characteristics of the participants (*n* = 14)

Code	Age	Sex	Education	Occupation	Risk behaviour of HIV infected	Being as HIV positive
P1	34	Male	Senior High School	Self‐employed	IDUs	Since 2001
P2	41	Female	Senior High school	Self‐employed	Heterosexual	Since 2015
P3	41	Female	College	Housewife	Heterosexual	Since 2007
P4	58	Female	Senior High School	Self‐employed	Heterosexual	Since 2011
P5	29	Female	Senior High School	Housewife	Heterosexual	Since 2017
P6	30	Male	Senior High school	Self‐employed	Homosexual	Since 2011
P7	37	Female	Senior High School	Housewife	Heterosexual	Since 2012
P8	21	Male	Senior High School	Unemployee	IDUs	Since 2014
P9	35	Male	College	Unemployee	IDUs	Since 2015
P10	29	Female	Senior High School	Self‐employed	Heterosexual	Since 2015
P11	46	Female	College	Self‐employed	Heterosexual	Since 2016
P12	37	Male	Junior High School	Self‐employed	Homosexual	Since 2012
P13	38	Male	Senior High School	Self‐employed	Homosexual	Since 2012
P14	34	Female	College	Housewife	Heterosexual	Since 2013

Abbreviations: IDUs, Injecting Drug Users; P, Participants.

In the study, we found five themes emerged from the data, namely free from stigma and discrimination, strengthen life spirit, have safe sexual practices, social support, and accessible and affordable healthcare services. The five themes were derived from 10 sub‐themes (Table 2).

**TABLE 2:** Theme of distribution

Themes	Major Categories	Minor Categories
Free from stigma and discrimination	Free from stigma	Isolated
		Insulted
	Free from discrimination	Hatred
		Refused treatment
Strengthen the life spirit	Attention from others	Understanding
		Motivation
		Spirit
	Repent and back to the God	God
Safe sexual practices	Change the risky sexual behaviour	Reducing the risk of transmission
		Secure
	Responsibility	Take care of a partner
Social support	Family	Mother's support
		Father's support
		Wife's support
		Children's support
	Community	Community acceptance
		Emotional support
		Financial and job opportunity
Accessible and affordable health care services	Easier access to hospital	Care
		Treatment
	Equal in distribution of health services	Incomplete
		Rural area

### 3.1 Free from stigma and discrimination

All the participants stated that stigma and society discrimination against PLWH was still prevalent. As a consequence, some preferred to hide their HIV status from the surrounding people in family and community. This theme was identified through the subthemes of free from stigma and discriminations.

(1). Free from stigma. Participants received a stigma like being placed on bed in the corner of the ward and different from other patients. In addition, participants also expressed insults from other people both in the community and healthcare settings which might affect their need. In addition, the participant also hopes not to be treated differently like other people.

"… I recalled when my son was hospitalized. The nurses said my son was placed on a bed in the corner of the ward. That was a place for B20 patients (B20 was a code for HIV positive patients). Anytime a nurse comes to change the sheet, she wears full body protection such as gown, face mask, and double handschoen. I felt sad, I thought the health workers shouldn’t act like that, because my son was HIV positive. But I could not complain. I hope my son is not treated differently…” [the participant’s facial expression looked sad while telling the situation] (P3. Interview).

(2). Free from discrimination. Participants received discrimination treated as hated and refused treatment. In the interview, it was found that the patient expects not to be discredited.

"I wondered when the doctor asked me to be discharged from the hospital as soon as possible, while my health was not fully recovered at the time. I still had fever and difficulty in breathing. However, I followed the doctor decision, then three days later after being discharged, my health condition became worse, and I was readmitted and placed in the isolation room. When I asked the doctor "why was I placed in this isolation room?" She said "we are sorry; you are placed in this room because your disease is dangerous". I was immediately shocked. However, I hope not to be discriminated against and to get still treated like other patient…” (P3.FGD).

### 3.2 Strengthen the life spirit

Almost all the participants are aware that HIV has been known as a chronic manageable disease. They realized that the presentation of the virus in their body has threaten their health and put them as stigmatized group of people. Furthermore, the presentation of chronic disease symptoms, poor psychosocial conditions, and long‐term treatment, sometimes made them frustrated and hopeless. However, they learned from heath care workers as well as their fellow that there is still hoped to live longer and healthy by following the healthcare workers advises. This theme was identified through the subthemes of attention and repent.

(1). Attention from others. Psychological reinforcement can have a positive influence on PLWH to live life well. PLWH setting experiences discrimination. Thus, they need understanding, motivation, and sprit from the closest people and the community.

"So… the first time I knew my HIV status, it was really shocking, and I was hopeless. I imagined how the rest of my life would be terrible and hard…at that time, I really needed someone who understood my feelings, to raise my spirit and motivate me to keep life” (P2. Interview).

(2). Repent and back to the God. In this study context, participants mostly perceived that the spirit of life was associated with their spirituality particularly believing in God. In fact, spirituality was practically defined as the main source of energy or power of life.

“…by believing in God (Allah), I am sure that He will help me. It is a reminder from God that I have to realize. I have to get closer to God and much gratitude for the rest of my life and all things given to me…by doing that, my heart is calm and I have more spirit to live, to take medicine, and to make sure that everything is all right” (P7. Interview).

### 3.3 Safe sexual practices

The need to practice safe sex was conveyed by both male and female participants. After knowing their HIV status, they were aware about the risk of the transmission through sexual intercourse. In fact, some of the participants contracted the disease through unsafe sexual relations with one or multiple partners. This theme was identified through the subthemes of change sexual behaviour and responsibility.

(1). Change the risky sexual behaviour. Participant shared a thought and advised others who still engage in such risky behaviour to be able to change these behaviours and reduce the risk of HIV transmission. In addition, using condoms during sexual intercourse can provide security.

“…if they (who have risky behavior) want to be healthy, they have to be aware of their health status by visiting a doctor, be faithful with one sexual partner, and use condoms while sexual intercourse. Please stop that risky sexual behavior… I always remind them to change their behavior especially those who are already infected not to spread the transmission…” (P10. FGD).

(2). Responsibility. The participant who is married to a negative spouse was also concerned about the risk of HIV transmission. Therefore, the participant attempted to follow safe sexual measures by using condom.

“…Before getting married to my wife, I have explained to her that I have this disease, and unpredictably she accepted… However, as my responsibility to my beloved wife, I always use condom when we have sexual relation. Praise to God, my wife has been tested and the result was negative…so right now we have no problem, and I will try my best to take care of my partner…” (P1. Interview)

### 3.4 Social supports

The participants acknowledged the importance of family and community support when they are in sickness condition. In fact, family is considered as the closest ones who have strong emotional connection. Within a family, the members commonly share happiness, responsibility, care, love, sadness, and grieving together. Apart from that, support from the community can also improve well‐being. This theme could be divided into sub‐theme of family and community support.

(1). Family supports. Families can provide the much‐needed support by participants. In fact, the family is still the participant's first helpers when experiencing a weak condition. Family support can come from mothers, fathers, wives, and children.

“…the main things that I need from the family is direct support, motivation, comfort, and other resources in order to be strong in facing this disease and encouragement that I can fight the disease. My parents and wives always support me” (P.4. Interview).

(2). Community supports. Community support that received including acceptance from surrounding people, emotional support, financial and job opportunity, as well as other available community resources. It can improve the participant well‐being.

“…The nurse in the clinic introduced me to a peer group support. They supported me to get involved with their activities such as weekly meeting, counselling, information sharing, fund raising, and social campaign to raise public awareness toward HIV/AIDS. They also helped me when I have to visit the clinic for blood checking…I got much support from them, so I did not feel alone, and I have job opportunity in my community” (P.9. Interview).

### 3.5 Accessible and affordable healthcare services

In this study, it was found that the participants expected health services and access that were easily accessible. This is because the location of health services such as hospitals that are far from home. In addition, the complicated and long process became a problem for PLHW to obtain medicine. They must go through several health facilities to get a reference letter to the hospital, and it spends a lot of money. The gap in the distribution of health facilities is also a concern for PLWH to get medicine. This theme was identified through the subthemes of easier access to hospital and equal in distribution of health services.

(1). Easier access to hospital. The participant hopes that access to health services in hospitals will be easier. This is because participants have to go through complicated procedures to get treatment and care at the hospital.

“…We wished that access to hospital services was made easier. However, the procedure to get health services was quite complicated for us. We have to go to community health center or primary clinic to obtain a reference letter. Then we have to visit the secondary hospital. Meanwhile, not all secondary hospital offers HIV treatment, therefore we have to visit tertiary level hospital. Actually, we prefer to come directly to this hospital, which is tertiary. If we have to go to multi‐level of health care service, we have to pay transportation fee” (P3. Interview).

(2). Equal in distribution of health services. Some participants were concerned about the need of care continuity for PLWH because they need long treatment and special care. Also, the equal in distribution of health care between urban and rural area should be promoted.

“…PLWH should be assisted, we need care for a long life, need to take medicine for a long life, checking CD4, Viral Load, and others as well. Therefore, we have to be facilitated by health insurance, not only those who live in the city but also all those who live in rural area” (P7. Interview).

## 4 DISCUSSION

The results of this study showed that PLWH identified several needs based on their point of view as reflected in the themes. Also, being free from stigma and discrimination was found as the first concern among the participants, which needs to be addressed. Although the HIV/AIDS has been existing for a long time, PLWH has shown better appearance due to the efficacy of anti‐retroviral treatment (ART) (Narsai et al., 2016). Meanwhile, stigma and discrimination towards these people seemed not to have completely disappear due to many factors (Nursalam et al., 2021). A study examined HIV‐related stigma among PLWH in rural central China and found that they suffered from the burden of HIV‐related stigma at a moderate to high level (Li et al., 2018). Meanwhile, men who have sex with men (MSM) infected with HIV were reported to have experiences related to stigma and shame such as labelling as whore and dirty, stereotyping of promiscuity, and rejected by the community (Dubov et al., 2018).

Stigma has been found to have negative association with patients’ quality of life (Januraga et al., 2018). Also, in healthcare setting, stigma towards PLWH remained prevalent though its magnitude was not as big as in the beginning of HIV epidemic. The previous study found that majority of healthcare providers have stigmatized attitude at a moderate to high level (Zarei et al., 2015). Furthermore, feelings of fear was the main reason for avoiding care provision for PLWH (Stutterheim et al., 2014). A study conducted in Vietnam also reported that almost half of nurses showed attitudes of not willing to care for PLWH (Ishimaru et al., 2017). Furthermore, stigmatizing attitude among nurses in Indonesia was high (Waluyo et al., 2015). Meanwhile, religious beliefs, degree of involvement in religion, and affiliation of the workplace contributed significantly to stigmatizing attitudes towards PLWH and populations at risk for HIV. This stigma can hinder the success of treatment and care; therefore effective interventions are strongly needed (Reyes‐Estrada et al., 2015).

The need to strengthen life spirit has been identified by participants of this study as second theme. The participants used the term “spirit” which closed to spirituality which is rooted on the belief in God (Allah) according to Islamic perspectives. Furthermore, all the participants were Muslim; therefore it influenced their point of view towards life including health and illness. In Islamic perspective, Mohsen noted that spirituality can refer to an awareness and belief that the Almighty Allah (God) exists (Handayani et al., 2017). In fact, the connection between spirituality and health outcomes has been well documented in literature (Widyanthini et al., 2014). Religiosity and spirituality played an important role in coping with HIV (Pinho et al., 2017). Furthermore, spiritual coping has been proved to have prospective association with survival among HIV patients and need to strengthen spirituality to promote health and survival of PLWH (Roscoe & Hachey, 2020).

Also, practicing safe sex becomes a major concern among PLWH. In fact, the use of condom as a preventive measure to reduce HIV transmission between sexual partner has been stated in several studies (Mukandavire et al., 2016; Staras et al., 2013; Wang et al., 2015). A meta‐analysis study found that condom reduced HIV transmission by more than 70% when used consistently by HIV sero‐discordant couples (Prevention, 2017). Also, the use of condom was still contentious in Muslim society such as Indonesia. This usage was prohibited for extra marriage couples, and even condom campaigns were considered a driving factor for promiscuity or extra marriage sexual relationship. However, many Muslim leaders agreed to using condom as a preventive measure of HIV transmission among sero‐discordant marriage couples (Umeokonkwo et al., 2019).

People Living with Human Immunodeficiency Virus need support from family, community, as well as healthcare providers. Family is the primary environment where the patients live and find love, warmth, and support. Previous study found that family support with no or minimal discrimination contributed to the increased quality of life among PLWH (Januraga et al., 2018). Although family is the primary source of support, social support is also important. This social support has been known as a determinant of health model (Alan R Lifson et al., 2013). Furthermore, it includes a set of social, economic, cultural, psychological, ethnical, and behavioural factors that influence health. Previous study documented that social support correlated with a lower proportion of depression (Matsumoto et al., 2017); therefore it could be seen as a key protective factor against depression in PLWH.

Access to affordable healthcare service is also a concern among PLWH since their health is susceptible due to the virus progression in their body. The remaining significant number of PLWH was loss to follow‐up (Handayani et al., 2017; Haroen et al., 2017; Widyanthini et al., 2014), and lower percentage of virally suppression (Januraga et al., 2018) was due to barrier to access healthcare services. Also, Previous study reported some inhibiting factors for PLWH in accessing healthcare services, which include stigma, long distances, lack of confidentiality, financial constraints, multiple appointments (Ayon et al., 2018). Furthermore, they include unfamiliarity with health facilities, disconnection in communication with healthcare providers, and lack of understanding regarding women's needs. These barriers were more apparent for sub‐risk group population such as homosexual, transgender, and PLWH with disabilities (Haroen et al., 2017). Once the barriers were not overcoming, the patients would come to the clinic with late presentation which could influence their health outcomes. This late presentation refers to individuals showing up for HIV care with a CD4 count below 350 cells/μl or with an AIDS‐defining event (Roscoe & Hachey, 2020). Furthermore, late presentation is associated with increased patient morbidity and mortality, healthcare costs, and risk of onward transmission by those who are unaware of their status. Finally, late presentation limits the effectiveness of all subsequent steps in the cascade of HIV care. A review study offered the possibility of home‐based care intervention to overcome the missing link of health care for PLWH (Larki & Roudsari, 2020).

The main strength of the present study is the ability to uncover healthcare need of PLWH from participants’ perspectives especially in Indonesian cultural context. The results of this study also provide rich information about the experience of PLWH in their daily life. Many of them have had to struggle with stigma and discrimination in various sectors such as family, workplace, and society, strengthening for living, support from the community, good and safe sexual education for PLHW, and easy access to treatment. Those basics are needed by PLHW. However, we realized that this study has limitation. We only explored the data from the participant's point of view (PLWH). Thus, broader and diverse data about healthcare needs among PLWH will be obtained by involving families and health service providers.

## 5 CONCLUSIONS

Although HIV epidemic has been lasting since decades ago, the disease remains prevalent in the community. Meanwhile, PLWH have their perception regarding the needs of healthcare services. This study found five themes that reflected the needs of PLWH regarding healthcare services. It is suggested that having a better understanding about their needs, and incorporated into nursing interventions would keep the patients engaged with the health care. Therefore, their treatment progression would be easily monitored and improved. The final treatment goal is to suppress the virus and improve quality of life. Further study is needed to develop and test the models of effective nursing intervention to address the need of PLWH, so comprehensive continuum of care for PLWH can be assured.

## CONFLICT OF INTEREST

The authors declared that they have no conflict of interest.

## AUTHOR CONTRIBUTIONS

KI, LR, and YKH designed the study. KI, SK and TS collected the data. KI, LR and YKH analysed the data. KI, LR, YKH, SK, and TS prepared the manuscript. All authors approved and contributed to the final version for submission.

## Data Availability

The data set that support the findings of this study are available from the corresponding author, [KI], upon reasonable request. The data are not publicly available due to privacy or ethical restrictions.
